# A Case Report of Normal Pressure Hydrocephalus Developing After COVID-19 Infection

**DOI:** 10.7759/cureus.33753

**Published:** 2023-01-13

**Authors:** Faraz Badar, Muhammad Azaz I Cheema, Asma Iftikhar

**Affiliations:** 1 Internal Medicine, Northwell Health at Mather Hospital, Port Jefferson, USA; 2 Pulmonary and Critical Care Medicine, Northwell Health at Mather Hospital, Port Jefferson, USA

**Keywords:** ventriculoperitoneal shunts, shunt, external ventricular drain (evd), covid 19, normal-pressure hydrocephalus

## Abstract

Severe acute respiratory syndrome coronavirus 2 (SARS-CoV-2 virus) has been reported to cause significant injury to the central nervous system (CNS). Herein, we describe the case of a 48-year-old male with a past medical history of attention-deficit/hyperactivity disorder (ADHD), hypertension, and hyperlipidemia who developed typical symptomatology of normal pressure hydrocephalus (NPH) with cognitive impairment, gait dysfunction, and urinary incontinence after a mild coronavirus disease (COVID-19) infection. The diagnosis was confirmed by imaging and lumbar puncture (LP). The patient was treated with a ventriculoperitoneal (VP) shunt placed by neurosurgery and had a complete recovery.

Despite increasing reports of neurological manifestations of COVID-19 infection, the mechanism of such pathology is still not well understood. Hypotheses include viral invasion of the CNS either through the nasopharynx and olfactory epithelium or directly through the blood brain barrier.

## Introduction

The coronavirus 2019 (COVID-19) pandemic caused by the contagious severe acute respiratory syndrome coronavirus 2 (SARS-CoV-2) virus has been the biggest health crisis of the last century [1]. The primary mode of viral transmission is airborne [2]. Most commonly causing upper and lower respiratory tract infections, the SARS-CoV-2 virus affects a wide variety of body organs, including but not limited to the cardiovascular system, gastrointestinal (GI) tract, immune system, and renal system [3,4]. The most common spectrum of infection ranges from mild symptoms of fever, fatigue, shortness of breath, cough, myalgias, rhinorrhea, sore throat, and diarrhea to severe symptoms of respiratory failure, pneumonia, kidney injury, thromboembolism, and septic shock [4]. Some studies report that more than 50% of hospitalized COVID-19 patients develop neurological manifestations [5,6]. The involvement of the CNS is poorly understood however medical literature on this topic is rapidly expanding [7-13]. Along these lines, we present the case of a 48-year-old male who developed normal pressure hydrocephalus (NPH) shortly after a COVID-19 infection. As per our literature review, there has been only one other similar case report published about NPH associated with COVID-19 infection [14].

## Case presentation

A 48-year-old male with a past medical history of attention-deficit/hyperactivity disorder (ADHD), hypertension, and hyperlipidemia presented with ongoing headaches, vertigo, gait instability, lethargy, confusion, blurry vision changes as well as nausea and vomiting for two and a half months. Symptoms initially started with headaches, which were localized to the forehead, pressure-like in quality, 7/10 in intensity, non-radiating, aggravated by movement, and without relieving factors. The patient had difficulty walking and maintaining balance but no falls or lower extremity paraesthesias. There was mild confusion, which was a change from baseline but no hallucinations or delusions were reported.

The patient was seen on an outpatient basis by both neurosurgery and neurology for these ongoing symptoms and was reportedly diagnosed with mild hydrocephalus. He underwent outpatient magnetic resonance imaging (MRI) of the brain remarkable for slightly enlarged ventricles. An ophthalmologic examination to rule out increased intracranial pressure did not show signs of papilledema. A lumbar puncture showed normal opening pressure of 10 cmH2O and the cerebrospinal fluid (CSF) studies, including cell count, protein and glucose levels were also normal. The patient also had a normal electroencephalogram (EEG).

Of note, the patient had a COVID-19 infection three months prior confirmed on outpatient polymerase chain reaction (PCR) testing. His symptoms were mild and confined to the upper respiratory tract and did not require anti-viral, corticosteroid, or supplemental oxygen therapy. He had not received any vaccinations for COVID-19. The patient’s mentation and lethargy kept worsening for a few days, prompting his wife to bring him in for hospitalization. He also developed urinary incontinence requiring Foley catheter insertion and had poor oral intake due to nausea and vomiting.

On neurological exam in the hospital, the patient was awake, alert, and oriented to person, time, and partially to place (he knew he was in a hospital but was unsure of which one). His speech was clear and fluent, and pupils were equally round and reactive to light. Extraocular movements revealed diplopia and horizontal nystagmus on bilateral gaze. Visual fields were intact. Facial strength and sensation were symmetric and intact, hearing was intact to finger rub bilaterally, and tongue was midline with symmetric palate elevation. Shoulder shrug was symmetric. Range of motion was complete and strength grading was 5/5 in bilateral upper and lower extremities. There was no pronator drift. Finger-nose testing showed mild left greater than right dysmetria. He also had mild bilateral upper extremity essential tremor. No bradykinesia or cogwheel rigidity was noted. Plantar reflex showed upgoing toes bilaterally. The gait exam showed postural instability with short, slow, and magnetic steps. The rest of the physical exam, including vital signs, was unremarkable. 

**Figure 1:**
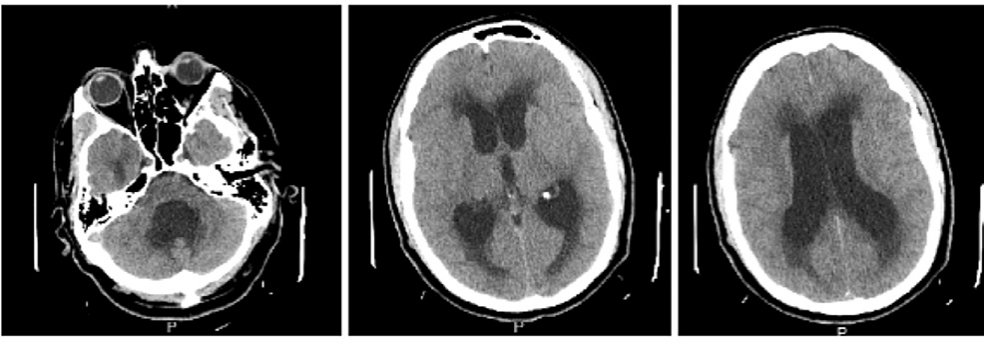
Moderate ventriculomegaly affecting all four ventricles, indicative of communicating hydrocephalus

CT scan of the brain on admission was positive for worsening hydrocephalus; moderate ventriculomegaly (worsened compared to prior outpatient CT) affecting the lateral, third, and fourth ventricles, development of periventricular hypodensity suggestive of transependymal flow of CSF and dysgenesis of the corpus callosum (Figure 1).

MRI scan of the cervical spine demonstrated increased dilatation of the fourth ventricle with mild periventricular T2 signal hyperintensity likely related to transependymal edema. Other chronic findings included cervical spondylosis and spondylolisthesis. Table 1 lists the laboratory workup. PCR testing for COVID-19 was negative on admission.

**Table 1:** Laboratory parameters WBC: White Blood Cells, INR: International Normalized Ratio, PT: Prothrombin Time, PTT: Partial Thromboplastin Time, BUN: Blood Urea Nitrogen, TSH; Thyroid Stimulating Hormone

Name	Result	Reference Range
WBC	10.8	3.5 - 10.8 K/ul
Hemoglobin	14.7	11.5 - 15.5 g/dl
Hematocrit	42.7	34.5 - 45.0 %
Platelet Count	352	150 - 400 K/ul
INR	1	0.8 - 1.2
PT	11.2	9.9 - 13.5 s
PTT	26.9	25.1 - 35.7 s
Sodium	141	136 - 145 mmol/L
Potassium	3.9	3.3 - 5.1 mmol/L
Chloride	102	98 - 107 mmol/L
Bicarbonate	30	22 - 29 mmol/L
BUN	16	8 - 23 mg/dl
Creatinine	1.0	0.7 - 1.2 mg/dl
Glucose	87	74 - 109 mg/dl
Magnesium	2	1.6 - 2.6 mg/dl
Phosphorus	3.7	2.5 - 4.5 mg/dl
TSH	0.97	0.27 - 4.2 uIU/ml

In light of hydrocephalus on brain imaging, the patient was transferred to the intensive care unit and neurosurgery placed an external ventricular drain (EVD) via a right frontal twist bur hole for decompression of the ventricular system as well as obtaining a CSF sample for appropriate studies to determine the cause. The patient’s clinical symptoms improved post EVD.

**Figure 2:**
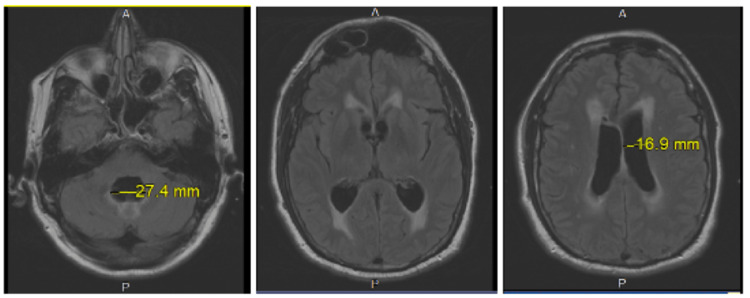
Improved ventriculomegaly and improved but persistent periventricular T2 hyperintensity consistent with improving the transependymal CSF flow

Post-procedure CT and MRI scans showed interval improvement in ventricle size as well. No mass lesion, abnormality, or obstruction was identified to explain the cause of hydrocephalus (Figure 2).

CSF studies, including cell count, protein, and glucose, were within normal limits. Neurosurgery went on to place a ventriculoperitoneal (VP) shunt three days after with the subsequent removal of EVD. An MRI lumbar spine prior to the laparoscopic performance of peritoneal catheter insertion did not reveal an intradural mass to explain the cause of hydrocephalus either. The patient made an unremarkable recovery in the hospital; he was downgraded to medical floors and ambulated with physical therapy. Butalbital/acetaminophen and topiramate were used for relief from any residual headaches. A Foley catheter was removed with a successful voiding trial. The patient was discharged to a rehabilitation facility and an outpatient shunt series on follow-up showed an intact VP shunt.

## Discussion

COVID-19 causes neurological manifestations, and this has been a subject of much interest due to increasing evidence and medical literature becoming available [7-13,15-16]. The mechanism proposed regarding CNS invasion by the SARS-CoV-2 virus is interaction with an angiotensin-converting enzyme 2 (ACE2) receptor on the olfactory epithelium in the nasopharynx. Neuro invasion directly from the bloodstream across the blood brain barrier has also been described [13,15]. Other mechanisms of CNS involvement include Systemic Inflammatory Response Syndrome (SIRS) and thromboembolism [16,17]. A varied range of neurological symptoms and manifestations associated with COVID-19 infection have been described. These include but are not limited to mild symptoms of headache, anosmia, and dysgeusia to severe clinical scenarios of encephalopathy, meningoencephalitis, Guillain-Barre Syndrome (GBS), ischemic stroke, intracerebral hemorrhage, seizures, myopathy and neuropathy [7-13].

The exact mechanism of nerve cell injury or damage by the virus is very poorly understood. Broadly, some initial hypotheses regarding the pathogenesis include direct viral invasion of neurons, damage to vascular cells resulting in hypoxia of the nervous system, or a proinflammatory state mimicking an autoimmune disorder [11,18,19].

We describe a rare and unique case of NPH developing in a patient after a COVID-19 infection. NPH is characterized by a classic clinical triad of cognitive impairment, gait dysfunction, and urinary incontinence [20]. It may be primary (also called idiopathic) or secondary to other disease states such as subarachnoid hemorrhage, meningitis, intracranial neoplasm, brain surgery, or traumatic brain injury [21]. In our literature review, we only came across one other case report describing a similar case of NPH associated with COVID-19 infection [14]. This case by Zubair et al. had very similar presenting symptoms with resolution after drainage of CSF. However, their patient had an earlier hospitalization due to hypoxia, requiring nasal cannula oxygen, whereas our patient did not require admission for the initial COVID-19 infection. Interestingly, a recent case of obstructive hydrocephalus with raised ICP after COVID-19 has been reported as well [22]. Occlusion of the fourth ventricular outlet secondary to altered CSF flow and arachnoid web formation from viral inflammation is the proposed hypothesis for this case.

Our patient lacked any comorbidities predisposing him to a disease process, such as NPH, and he developed symptoms of cognitive impairment, gait dysfunction, and urinary incontinence within two weeks of COVID-19 infection. We were unable to confirm COVID-19 as the causative agent due to technical difficulties and a lack of laboratory facilities to perform PCR testing on CSF samples. Despite this, our patient’s clinical scenario, as well as the aforementioned case report, suggests a temporal association between COVID-19 infection and NPH. Like typical NPH, our patient had cognitive impairment, gait dysfunction, urinary incontinence, and MRI imaging demonstrative of ventriculomegaly. VP shunt placement is one of the standard treatments for NPH, and our patient demonstrated significant symptomatic improvement post-procedure. This highlights that despite the association with a viral infection, the diagnostic and therapeutic approach for managing such a case of NPH remains unchanged.

## Conclusions

To summarize, we report an unusual case of NPH presenting as a possible complication of COVID-19 infection. Although primarily known for respiratory manifestations, neurological disorders secondary to COVID-19 are an emerging clinical problem. Pathogenesis regarding the neurological features of COVID-19 remains unclear. Input from neurologists and further research is needed for a better understanding of the impact and outcomes of neurological disease from COVID-19 infection. Management guidelines are yet to be defined, therefore, prompt identification of morphology and symptoms in cases such as ours can help guide diagnosis and treatment.
